# Quantitative Trait Locus Mapping and Identification of Candidate Genes Controlling Flowering Time in *Brassica napus* L.

**DOI:** 10.3389/fpls.2020.626205

**Published:** 2021-02-03

**Authors:** Yu Xu, Bingbing Zhang, Ning Ma, Xia Liu, Mengfan Qin, Yan Zhang, Kai Wang, Na Guo, Kaifeng Zuo, Xiang Liu, Miao Zhang, Zhen Huang, Aixia Xu

**Affiliations:** ^1^State Key Laboratory of Crop Stress Biology for Arid Areas/College of Agronomy, Northwest A&F University, Yangling, China; ^2^Institute of Vegetables and Flowers, Jiangxi Academy of Agricultural Sciences, Nanchang, China; ^3^Market Supervision Administration, Yanchi, China

**Keywords:** Brassica napus, flowering time, SLAF-seq, QTL, candidate gene

## Abstract

Flowering time plays a vital role in determining the life-cycle period, yield, and seed quality of rapeseed (*Brassica napus* L.) in certain environments. Quantitative trait locus (QTL) mapping to identify the genetic architecture of genes controlling flowering time helps accelerate the early maturity breeding process. In this study, simple sequence repeats (SSR) and specific-locus amplified fragment sequencing (SLAF-seq) technologies were adopted to map the QTLs for flowering time in four environments. As a result, three target intervals, *FTA09*, *FTA10*, and *FTC05* were identified. Among this, *FTA09* was considered as a novel interval, *FTA10* and *FTC05* as stable regions. Based on the parental re-sequencing data, 7,022 single nucleotide polymorphisms (SNPs) and 2,195 insertion-deletions (InDels) between the two parents were identified in these three target regions. A total of 186 genes possessed genetic variations in these intervals, 14 of which were related to flowering time involved in photoperiod, circadian clock, vernalization, and gibberellin pathways. Six InDel markers linked to flowering time were developed in the three target intervals, indicating that the results were credible in this study. These results laid a good foundation for further genetic studies on flowering-time regulation in *B. napus* L.

## Introduction

Rapeseed (*Brassica napus* L.), one of the most important oilseed crops, is adaptive to different geographical environments by adjusting flowering time (FT; Shah et al., 2018). Flowering time of rapeseed plays a critical role in determining the life-cycle period, yield, and seed quality. Therefore, understanding these regulatory factors of FT not only assists in improving yield and adaption but also contributes to accelerating the early maturity breeding process.

*Brassica napus* (genome AACC, 2*n* = 38) is an allopolyploid species which is originated from spontaneous interspecific hybridization events between *Brassica rapa* (genome AA, 2*n* = 20) and *Brassica oleracea* (genome CC, 2*n* = 18) during the last few thousand years (Snowdon et al., 2007). Random gene loss and genetic drift have differentiated both the composition of the segments and the orthologous gene copies within the A and C genome (Cheung et al., 2009). The resultant genome structure shows that there could be two to six homologs for some genes in *B. napus* (Parkin et al., 2010). The multiple relevant gene copies hamper the study of some gene expressions and limit the understanding of trait-controlling mechanisms, especially for quality traits.

Flowering time is a complex and environmentally responsive trait that is controlled by endogenous genes and environmental factors (Gaudinier and Blackman, 2020). Quantitative trait locus (QTL) mapping is a powerful tool for revealing the genetic architecture of complex traits (Mauricio, 2001). Many QTLs for FT were determined in *B. napus* (Wang et al., 2016; Xu et al., 2016; Li et al., 2018a; Shah et al., 2018; Jian et al., 2019). Genetic linkage maps based on DNA markers have become the predominant tools for probing QTLs (Ott et al., 2015; Song et al., 2017), of which many simple sequence repeat (SSR) markers have been used in developing genetic linkage maps for *B. napus* (Li et al., 2013). However, the low polymorphism rate of SSR in *B. napus* makes it difficult to build a saturated genetic map, which has limited the application of the genetic map in DNA marker-assisted selection.

With the rapid development and application of high-throughput sequencing technologies, the single nucleotide polymorphism (SNP) markers have become popular because of its enriched polymorphism across the whole genome, which is conducive to construct high-quality genetic maps, providing precise and complete information about the locations of QTLs or genes. So far, several QTLs for FT have been detected in rapeseed using the Illumina Infinium *Brassica* 60 K SNP array (Liu et al., 2013; Jian et al., 2019) and genome-wide association studies (Zhou et al., 2018). Based on these approaches, some flowering-time candidate genes were identified in oilseed rape (Xu et al., 2016; Shen et al., 2018; Wu et al., 2019). For example, *BnaA02g02560* and *BnaA02g08490* were proposed as candidate genes associated with plant height and FT (Shen et al., 2018). One hundred and fifty-one candidate genes corresponding to 95 homologous genes in *Arabidopsis thaliana* related to flowering were identified by genome-wide QTL analysis in *B. napus*, including *BnaC03g32910D*, *BnaA02g12130D*, and *BnaA03g13630D* (Li et al., 2018a).

Furthermore, several other sequencing technologies have also been developed for discovering SNPs, such as specific locus amplified fragment sequencing (SLAF-seq), which is an efficient solution for large-scale genotyping at low cost (Sun et al., 2013). Recently, SLAF-seq has been applied in many plant species for developing high-density genetic maps and QTL mapping. For example, a high-density genetic map with 3,326 genetic markers was developed using SLAF-seq, and 15 significant QTLs for floret flowering time were detected (Wang et al., 2020). A total of 13 stable QTLs associated with six cottonseed quality traits were detected based on a high-resolution genetic map with 7,033 SNP loci obtained by SLAF-seq (Ali et al., 2018). In *B. napus*, 100 genes associated with plant resistance, vernalization response, and signal transduction were detected in SNP-rich genomic regions among 300 rapeseed accessions using SLAF-seq (Zhou et al., 2017). These results show that SLAF-seq is a powerful technique for effective linkage map construction and QTL analysis. However, SLAF-seq has not been fully applied in revealing the genetic basis of flowering time in *B. napus*.

The genetic architecture and molecular basis for the flowering time have been well described in the model plant *A. thaliana* (Fornara et al., 2010; Bouché et al., 2016). However, in *B. napus*, the genetic mechanism of flowering time remains poorly understood. To further elucidate QTL regions and candidate genes of flowering time in *B. napus*, the purposes of this study aim to (1) construct a high-density genetic map using two F_2_ populations; (2) identify the QTLs related to flowering time in multiple environments based on SSR and SLAF-seq; and (3) obtain the novel flowering time sites and genes responsible for flowering time. This study will lay a good foundation for studying the molecular mechanism of flowering time and early maturity breeding in rapeseed.

## Materials and Methods

### Plant Materials and Field Experiment

Two F_2_ populations derived from GW (late flowering) and DZ (early flowering) were used for genetic map construction. One F_2_ population comprising 170 individuals (F_2a_ population) and two parental plants used for SSR genetic map construction were planted in the experimental field of Northwest Agriculture and Forestry University in Yangling, Shaanxi, China (34.28’N, 108.07′E) in 2016 (YL16), and a total of 170 F_2:3_ families were sown at Yangling and Doukou Experimental Station (Sanyuan, Shaanxi, China; 34.62’N, 108.93′E) in 2017 (YL17 and SY17) by selfing each F_2a_ plant. The other F_2_ population including 80 individuals (F_2b_ population) and two parental plants were grown in Yangling in 2018 (YL18) for SLAF-seq.

### Phenotypic Evaluation and Statistical Analysis

Flowering time refers to the period from sowing the first open flower. FT was investigated daily during the whole flowering period for both parents, F_2_ and F_2:3_ lines in 2016, 2017, and 2018. A total of 20 plants for each parent were investigated for the flowering date every year, and FT was recorded for each F_2:3_ family (15–20 individuals) when half of the plants were initiated flowering (Li et al., 2018a). In addition, budding time (BUT) and bolting time (BOT) were recorded as the days from sowing to the emergence of buds and the achievement of a 3-cm-high main flower stalk, respectively, for F_2b_ plants in YL18 (Li et al., 2009a). Statistical analyses of all indicators were carried out with SPSS 20.0. Correlation analysis among the indicators (FT, BOT, and BUT) for the F_2b_ population was performed using Pearson’s test.

The broad-sense heritability (*h*^2^) of FT in YL16, YL17, and SY17 was implemented using the lmerTest package in R.^1^ The random effects were estimated using the best linear unbiased prediction (BLUP) by the “lmer” function executed in the R package *lme4*.

### DNA Extraction

At the flowering stage, fresh young leaves of F_2a_ and F_2b_ populations and parents were collected, immediately frozen in liquid nitrogen, and stored in a −80°C freezer. Total genomic DNA was extracted from the fresh leaves according to a modified cetyltrimethylammonium bromide method (Zhang et al., 2005). The quality and concentration of DNA were assessed by electrophoresis on 1% agarose gels and a NanoDrop one spectrophotometer (Thermo Scientific, United States).

### SSR Linkage Map Construction and QTL Mapping

Simple sequence repeat primers were obtained from public sources, including the series of BrGMS and BnGMS (Cheng et al., 2009; Xu et al., 2010), the database on http://ukcrop.net/perl/ace/search/BrassicaDB (Lowe et al., 2004), https://www.brassica.info/resource/markers/ssr-exchange/SSRinfo.htm, and the electronic Supplementary Material of Piquemal et al. (2005). First, the SSR primers were used to screen the two parents GW and DZ, and the polymorphic markers were used to genotype the 170 F_2a_ individuals grown in YL16. The polymerase chain reaction (PCR) products were detected by silver staining.

The SSR genetic linkage map of the F_2a_ population was conducted with JoinMap 4.0 (Van Ooijen, 2006). A minimum logarithm of the odds (LOD) score of 4.0 was used to group loci into linkage groups (LGs). QTLs based on the SSR linkage map (as SSR-based QTLs) were investigated using WinQTLCart 2.5 software.^2^ The window size, the working speed, and the control marker number were set at as 10, 1, and 5 cM, respectively. A standard model for composite interval mapping (CIM) was used and a log-likelihood of 2.5 was set as the threshold of a significant SSR-based QTL. The nomenclature of QTLs was referred to Quijada et al. (2006), the initial letter “q” followed by the abbreviation of the trait, the environment name, a “−,” and the corresponding LG or chromosome (e.g., qFTYL-16). The linkage genetic map and QTLs were graphically showed using MapChart 2.2 (Voorrips, 2002).

The corresponding chromosomes and physical positions of SSR-based QTLs were confirmed by the alignment with the Darmor-*bzh* genome (Chalhoub et al., 2014) using the BLAST tool.

### SLAF Sequencing and Genotyping

To build a high-resolution genetic map (HRGM), whole-genome re-sequencing for two parents (GW and DZ) and a SLAF-seq for 80 F_2b_ individuals were carried out at the Biomarker Technologies Corporation (Beijing, China). The reference genome of *B. napus*^3^ was used to predict digestion, and two enzymes (HaeIII+Hpy166II, New England Biolabs, United States) were chosen to digest the genomic DNA. Restriction fragments ranging from 464 to 514 bp in size were defined as SLAF markers, and then, high-throughput sequencing was carried out using an Illumina Hiseq™ 2500 system (Illumina, Inc., San Diego, CA, United States). The low-quality reads (quality score <20e) were initially filtered out, and then 5 bp terminal sites were trimmed to yield high-quality reads. Clean reads were mapped to the reference genome using Burrows-Wheeler-Aligner (Li and Durbin, 2009).

The reads mapped to the same genome position with over 90% identity were grouped to one SLAF locus. SNP loci of each SLAF locus were detected between the two parents, and SLAFs with more than three SNPs were filtered out by GATK software and Samtools (Li et al., 2009b). Only SLAFs with two to four alleles were identified as polymorphic and considered as potential markers. All polymorphic SLAF loci showing consistent both in the parental and offspring SNP loci were classified into eight types (ab × cd, ef × eg, ab × cc, cc × ab, hk × hk, lm × ll, nn × np, and aa × bb) according to the genotype encoding rule. Since the parents were homozygous, only the aa × bb polymorphisms were used for analysis. After filtering the low-quality SNPs with a sequencing depth of ≤5-fold in parents or a complete degree of ≤60%, the remaining SNPs were used for constructing the linkage map.

### A High-Resolution Genetic Map Construction and QTL Analysis

Polymorphic SLAF markers were aligned with the reference genome and mapped onto 19 chromosomes of *B. napus*, and each chromosome was an LG. The modified logarithm of odds (MLOD) scores between markers were calculated and filtered with MLOD values of less than 5. The HighMap software (Liu et al., 2014) was used to analyze the markers in LGs and calculate the map distances. Besides, SMOOTH (van Os et al., 2005) was applied to correct errors based on the parental contribution of genotypes, and a k-nearest neighbor algorithm was applied to impute missing genotypes. Skewed markers were then mapped by applying a multipoint method of maximum likelihood. Map distances were estimated using the Kosambi mapping function in centimorgan (cM).

Basing on the HRGM, QTLs (as SLAF-based QTLs) for FT, BUT, and BOT were detected using the QTL IciMapping software (Meng et al., 2015), combing with the phenotype of the F_2b_ population planted in YL18. Composite Interval Mapping of ADDitive QTL (ICIM-ADD) method was exploited for SLAF-based QTLs. Parameters were set as the following: a step in 1 cM, probability in stepwise regression of 0.001, and LOD = 2.5.

### Integrating Linkage Maps and Identifying Co-localized Intervals

To determine the major fiducial intervals of flowering time in *B. napus*, the SSR markers in the SSR-based QTLs were used to detect genotypes of the F_2b_ population. Combining with the SNP markers of the F_2b_ plants, an integrated high-definition genetic map (IH-DGM) was constructed using the QTL IciMapping software (Meng et al., 2015), as the following steps. All markers were firstly grouped based on a threshold of LOD = 3.0. Secondly, the algorithm called nnTwoOpt was used to order markers. After ordering, each marker was rippled with a window of five markers and the rippling criteria of SARF for fine-tuning. Finally, the linkage map was output to detect the co-localized intervals.

On the integrated linkage groups, the co-localized intervals were identified by incorporating the loci of SSR- and SLAF-based QTLs according to the physical positions. Besides, in order to reduce the loss of the loci associated with FT in the HRGM, the regions repeatedly detected in the FT, BUT, and BOT traits in the F_2b_ population were also noted as co-localized intervals.

### QTL Comparison of Flowering Time Among Different Mapping Populations and Conditions

To appraise the co-localized intervals, previously reported loci and QTLs related to the flowering time of *B. napus* were collected (Wang et al., 2016; Xu et al., 2016; Wei et al., 2017; Li et al., 2018a,b; Shah et al., 2018; Shen et al., 2018; An et al., 2019; Jian et al., 2019; Raman et al., 2019; Wu et al., 2019; Song et al., 2020). Their physical positions (including the target intervals’) were aligned based on the same reference genome sequence of *B. napus* (Chalhoub et al., 2014). The corresponding loci that were from different populations and conditions were arranged using MapChart 2.2 software (Voorrips, 2002).

### Candidate Gene Analysis Within Target Intervals

In order to predict the candidate genes linked to FT, the SNPs and InDels in the target intervals were obtained from the re-sequencing data, and these genes with sequence variations between the two parents were found as hypothesized candidate genes. Putative functions of these genes were annotated based on BLAST search in four databases, including the protein family (Pfam) database^4^ InterPro database^5^ the Gene Ontology (GO)^6^ and the Kyoto Encyclopedia of Genes and Genomes (KEGG).^7^ Concurrently, flowering-time related genes (FTRG) of *A. thaliana* and their functions were collected from the Flowering Interactive Database (FLOR-ID; Bouché et al., 2016). By the annotations of the hypothesized candidate genes and the functions of FTRG in *A. thaliana*, these genes with similar functions or functional domains were listed as major candidate genes.

Based on the re-sequencing data, the genetic variations of the candidate genes between two parents were analyzed. The distributions of SNPs or InDels in the upstream (the 5 kb within the region of transcription start), intragenic region, and the downstream (the 5 kb within the region of transcription stop) were arranged by MapChart 2.2 (Voorrips, 2002).

### Development of InDel Markers in the Candidate Regions

To confirm the accuracy of the target regions, the genes containing over 10 bp InDel variations (deletion or insertion) within target intervals were selected to develop the InDel markers according to the re-sequence data of both parents. A total of 200-bp flanking sequences of the InDels were used to design the InDel primers with the Tm value of 55–59°C. These primers were used to amplify a small population, including the parents, 10 early-flowering and 10 late-flowering individuals.

A PCR was performed in a 20 μL volume containing 10 μL 2 × Rapid Taq Master Mix (Vazyme, Nanjing, China), 0.5 μL 10 μM forward and reverse primers each, 1 μL genomic DNA (100–300 ng), and 8 μL distilled water. Thermo cycling was started at 95°C for 3 min, followed by 35 cycles of 95°C for 15 s, 55°C for 15 s, and 72°C for 5 s, and a final extension at 72°C for 5 min. Electrophoresis was carried out in 3% agarose gel stained with Ultra GelRed Nucleic Acid Stain (Vazyme, Nanjing, China) for 30–60 min, and the banding patterns were visualized on a ChampGelTM 6000 system (Saizhi, Beijing, China) under UV light.

## Results

### Phenotypic Variations

The phenotypic variations for FT, BUT, and BOT of the parents, F_2_ and F_2:3_ in 2016, 2017, and 2018 are presented in Table 1 and Supplementary Figure S1. GW and DZ showed an extremely significant difference in the three indicators and different environments. In F_2_ and F_2:3_ populations, a wide range with an approximately continuous normal distribution and transgressive segregation were discovered for FT, BUT, and BOT in these environments. The results indicated that these traits were quantitative traits and the corresponding loci were presented in two parents. The broad-sense heritability (*h*^2^) value of FT was 0.80 across YL16, YL17, and SY17, which indicated that flowering time may be influenced to a certain extent by the environment.

**Table 1:** Statistical analysis of FT, BUT, and BOT for the F2 populations, F2:3 families, and their parents.

Trait (days)	Environ.	Parents		F_2_ populations				F_2:3_ families				*h*^2^^a^
		GW	DZ	Range	Mean ± SD	Skewness	Kurtosis	Range	Mean ± SD	Skewness	Kurtosis	
FT	YL16	196.64 ± 1.50	181.10 ± 1.26^**^	178-202	190.15 ± 4.71	0.04	0.17	-	-	-	-	0.80
	YL17	197.10 ± 1.80	182.10 ± 2.71^**^	-	-	-	-	180-194	187.61 ± 2.89	−0.2	−0.05	
	SY17	198.05 ± 1.88	182.05 ± 1.76^**^	-	-	-	-	183-200	189.92 ± 3.53	0.15	−0.02	
	YL18	197.10 ± 1.06	182.10 ± 0.98^**^	183-198	189.69 ± 3.5	−0.09	−0.12	-	-	-	-	-
BUT	YL18	174.82 ± 1.34	163.27 ± 1.09^**^	162-176	169.90 ± 2.88	−0.75	0.54	-	-	-	-	-
BOT	YL18	182.53 ± 0.79	167.44 ± 1.24^**^	165-183	174.08 ± 3.49	−0.32	0.25	-	-	-	-	-

BOT, bolting time; BUT, budding time; Environ., environment; FT, flowering time; SD, standard deviation; SY17, Sanyuan in 2017; YL16 and YL18, Yangling in 2016 and 2018.

a The broad-sense heritability value of FT across YL16, YL17, and SY17.

** Indicated the significance at the level of 1% among GW and DZ.

### SSR Genetic Map and SSR-Based QTLs for FT

A total of 1,030 pairs of SSR primers were used to screen the two parents, GW and DZ, of which, 119 pairs showed polymorphism. Subsequently, these polymorphic primers were used to screen the 170 F_2a_ individuals, among which, 107 pairs showed polymorphism with a polymorphic rate of 89.92%. A total of 160 polymorphic bands were amplified, 110 of which were grouped into 18 Linkage groups with a LOD threshold of 4.0 or 5.0 (Supplementary Table S1). The total length of the SSR genetic map was 900.33 cM with an average genetic distance of 8.5 cM between adjacent markers. The linkage map with the maximum SSR markers was 92.3 cM, but only eight markers on the longest linkage group (Supplementary Table S1). These results suggested that the SSR markers were sparsely populated.

Combining the SSR genetic linkage map with the flowering time in YL16, YL17, and SY17, three QTLs were detected in YL16, and two QTLs were identified in YL17 and SY17 (Supplementary Figure S2 and Table 2). The five QTLs were distributed across five linkage groups, including A02, A06, A07, A10, and C05, with an LOD value ranging from 2.94 to 4.57 (Table 2). Individual QTL explained 3.74–12.28% of the phenotypic variation (PV). Among these, *qFTYL16-5* and *qFTSY17-7* located on C05 and A10 accounted for 12.28 and 12.17% of the total PV, respectively. *qFTYL16-16* had positive effects on flowering time, and the positive effect alleles were mainly contributed by the male parent DZ. The remaining four QTLs (*qFTYL16-2*, *qFTYL16-5*, *qFTYL17-6*, and *qFTSY17-7*) had negative effects, and the positive effect alleles were mainly contributed by the female parent GW (Table 2).

**Table 2:** QTL for flowering time identified basing on SSR genetic map.

QTLs	LG	Chr.	Positions (cM)	Intervals	LOD	Effect^a^	PVE (%)	Lines and environment
*qFTYL16-2*	2	A06	58.81	BrGMS453 – BrGMs753	2.94	−2.00	3.74	F_2a_ population at Yangling in 2016
*qFTYL16-5*	5	C05	1.01	BnGMS309 – BnGMS327	3.94	−1.22	12.28	
*qFTYL16-16*	16	A07	15.41	BnGMS370	3.68	1.71	7.28	
*qFTYL17-6*	6	A02	28.91	BnGMS650 – BrGMS75	4.57	−1.23	4.20	F_2:3_ family at Yangling in 2017
*qFTSY17-7*	7	A10	19.01	BnGMS625 – BrGMs579	2.94	−0.98	12.17	F_2:3_ family at Sanyuan in 2017

Chr., chromosome; LG, linkage group; LOD, logarithm of odds; PVE, phenotypic variance explained.

a Positive and negative additive effects indicate that early flowering alleles are from DZ and GW, respectively.

### The High-Resolution Genetic Map Basing on SLAF-Seq

In order to construct a HRGM, we performed simplified genome sequencing on the F_2b_ population planted in YL18 and whole-genome re-sequencing for both parents. A total of 95.23 Gb data containing 378.88 Mb paired-end reads were generated. The average Q30 ratio of sequencing (based on a quality score of 30, 99% confidence) was 93.78%, and the average GC content was 39.91%. A total of 3,692,498 SNPs were obtained, of which, 2,417,208 were successfully encoded as polymorphic among parents and the F_2b_ population. These polymorphic SNPs were classified into eight types (ab × cd, ef × eg, ab × cc, cc × ab, hk × hk, lm × ll, nn × np, and aa × bb), and 1,098,307 SNPs belong to aa × bb with a homozygous genotype for both parents. Markers with less than 5x sequencing depth or more than 40% missing data were filtered. As a result, 3,356 SNP markers were retained for the genetic map construction, and 3,161 SNP markers were mapped on 19 chromosomes based on the *B. napus* reference genome using the HighMap software. The total length of the map was 2,195.02 cM with an average distance of 0.70 cM. The proportion of gaps of less than 5 cM was 98.73% (Supplementary Table S2 and Supplementary Figure S3A). Collinearity analysis between the genetic linkage map and the reference genome showed that most genetically mapped loci were collinear with their physical map locations on the 19 chromosomes (Supplementary Figure S3B). The high collinearity indicated that the gene annotation with QTL intervals was reliable.

### QTLs Related to Flowering Time Based on SLAF Genetic Map

**Figure 1:**
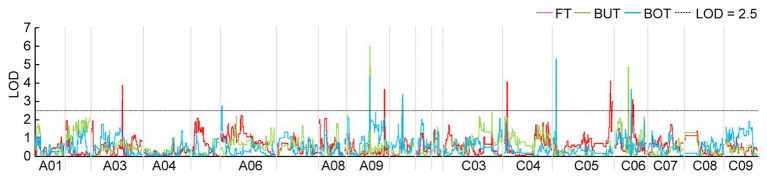
Significant quantitative trait loci (QTLs) for flowering time (FT), budding time (BUT), and bolting time (BOT) in the F_2b_ population assessed in Yangling in 2018 (YL18). The logarithm odds (LOD) threshold of 2.5 was used to declare the presence of QTL using the QTL IciMapping software. The colors of the identified QTLs are given in the figure. BOT, bolting time; BUT, budding time; FT, flowering time; YL18, the environment of Yangling in 2018.

Based on the HRGM and the traits associated with flowering time of the F_2b_ population planted in YL18, 14 QTLs were identified, including five for FT, four for BUT, and five for BOT, which explained 9.18–23.38% of the total phenotypic variance (PV) with LOD values ranging from 2.75 to 5.98. These QTLs were located on A03, A06, A09, A10, C04, C05, and C06 chromosomes (Figure 1 and Table 3). The four QTLs (*qBOTYL18-A10*, *qFTYL18-C6*, *qBUTYL18-C6*, and *qBOTYL18-C6*) accounted for the high PV that was over 20%. Among these, the three QTLs on C06 were closer with a genetic distance of 5.25 cM between each other (Table 3). Only *qFTYL18-A9* showed negative additive effects, and the early flowering genes were derived from the female GW. The rest of the QTLs had positive additive values, and the positive effects were from the male DZ (Table 3).

**Table 3:** QTLs for FT, BUT, and BOT of *Brassica napus* basing on HRGM^a^.

Traits	QTLs	Chromosome	Positions (cM)	Intervals	LOD	Effect^b^	PVE (%)
FT	*qFTYL18-A3*	A03	88.96	Marker234317 – Marker237645	3.86	2.09	18.79
	*qFTYL18-A9*	A09	143.42	Marker748838 – Marker749082	3.64	−1.46	13.16
	*qFTYL18-C4*	C04	7.50	Marker1143139 – Marker1143141	4.04	1.01	13.81
	*qFTYL18-C5*	C05	133.36	Marker1416161 – Marker1418915	4.10	1.75	13.46
	*qFTYL18-C6*	C06	51.67	Marker1473636 – Marker1473647	3.08	0.20	20.23
BUT	*qBUTYL18-A9*	A09	93.38	Marker718950 – Marker721850	5.98	0.26	10.67
	*qBUTYL18-A10*	A10	29.51	Marker798064 – Marker799287	3.18	1.09	11.87
	*qBUTYL18-C5*	C05	18.50	Marker1282614 – Marker1284391	4.15	1.60	18.03
	*qBUTYL18-C6*	C06	41.17	Marker1469639 – Marker1470029	4.85	0.07	21.79
BOT	*qBOTYL18-A6*	A06	3.38	Marker422869 – Marker422998	2.75	1.03	9.18
	*qBOTYL18-A9*	A09	93.38	Marker718950 – Marker721850	4.35	0.77	11.11
	*qBOTYL18-A10*	A10	33.17	Marker800510 – Marker800511	3.37	1.23	20.35
	*qBOTYL18-C5*	C05	18.50	Marker1282614 – Marker1284391	5.30	2.01	20.00
	*qBOTYL18-C6*	C06	46.42	Marker1472234 – Marker1472235	3.65	0.24	23.38

BUT, budding time; BOT, bolting time; LOD, logarithm of odds; PVE, phenotypic variance explained.

a The high-resolution genetic map was constructed by SLAF-seq using the F2b population.

b Positive and negative additive effects indicate that early flowering alleles are from DZ and GW, respectively.

### Co-localized QTLs Related to Flowering Time

**Figure 2:**
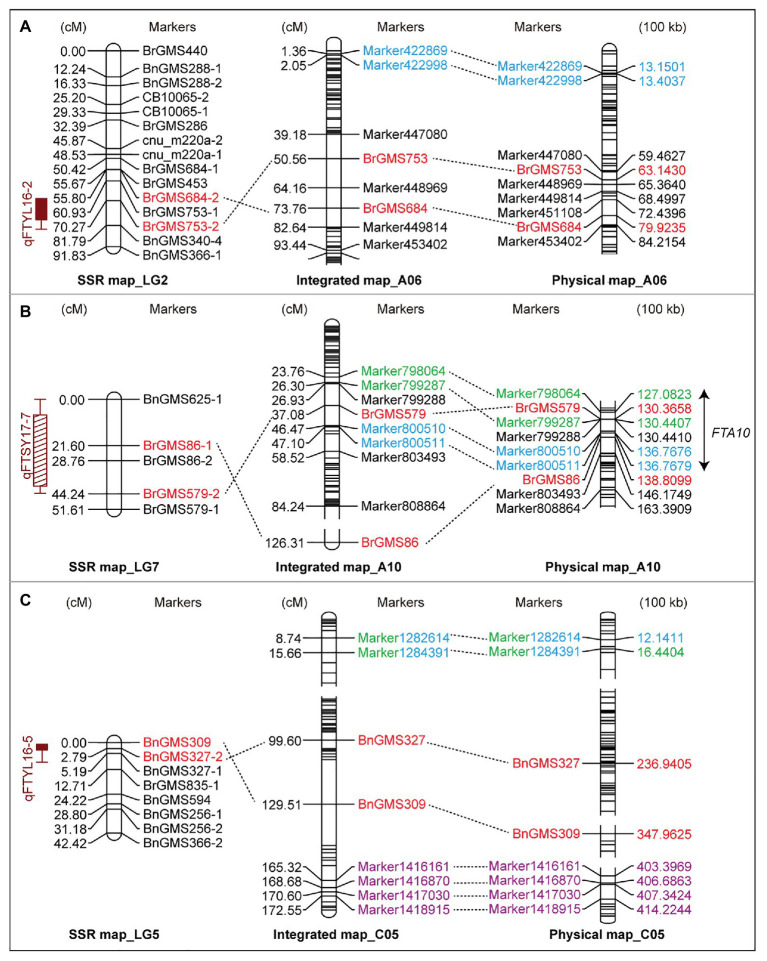
The integrated linkage groups by jointing the simple sequence repeat (SSR) and specific-locus amplified fragment (SLAF) markers. The SSR-based genetic map (left), the integrated genetic map (middle), and the aligned physical map (right) in A06 **(A)**, A10 **(B)**, and C05 **(C)** chromosomes are shown. Red markers represent the loci detected on the SSR-based map using the flowering time in YL16 and SY17. Blue, green, and purple markers label the positions identified by the BOT, BUT, and FT traits in YL18 on the high-resolution genetic map (HRGM), respectively. The same markers among these maps are connected by dotted lines. The double arrow marks the co-localized interval *FTA10* among these loci on A10. BOT, bolting time; BUT, budding time; FT, flowering; SY17, YL16, and YL18, the environments of Sanyuan in 2017, Yangling in 2016 and 2018.

According to the QTLs identified in the F_2a_ population, 11 SSR markers around the QTLs were adopted to screen the 80 F_2b_ individuals (Supplementary Table S3). The markers showing polymorphism were added into the high-resolution genetic map to construct an IH-DGM. In accordance with the genotyping results, six SSR markers from three SSR-based QTLs (*qFTYL16-2*, *qFTYL16-5*, and *qFTSY17-7*) were significantly linked with SLAF markers with an LOD score of >2.5. The six markers were mapped on A06 (BrgMS684 and BrgMS753), A10 (BrgMS579 and BrgMS86), and C05 (BnGMS309and BnGMS327) in the IH-DGM. The sequence comparison showed that there was a relatively high colinearity between these six SSR and SLAF markers (Figure 2 and Table 2).

On the three integrated linkage groups (A06, A10, and C05), there was a co-localized interval *FTA10* between SSR- and SLAF-based QTLs detected under two conditions (SY17 and YL18). *FTA10* included two SLAF-based QTLs (*qBUTYL18-A10* and *qBOTYL18-A10*) and an SSR marker from *qFTSY17-7*, locating on close physical sites, which indicated that *FTA10* was a stable QTL. On A06 and C05, there were no intersections among these QTLs identified in YL16, YL17, SY17, and YL18 (Figure 2).

In order to comprehensively detect the flowering time loci, the regions repeatedly detected for the FT, BUT, and BOT on HRGM were also retained as co-localized QTLs for further analysis. Two intervals (*FTA09* and *FTC05*) were identified (Table 4). Four SLAF-based QTLs (*qBUTYL18-A9*, *qBOTYL18-A9*, *qBUTYL18-C5*, and *qBOTYL18-C5*) located in these two intervals accounted for 10.67–20% of the total phenotypic variations with LOD scores from 4.15 to 5.98 (Table 3).

**Table 4:** Details of target intervals.

Target intervals	Chr.	Flanking markers	Physical location (Mb)	Genes NO.^a^	SNPs/Genes NO.^b^	InDels/Genes NO.^c^
*FTA09*	A09	Marker718950 – Marker721850	21.34–22.10	104	2,759/53	938/47
*FTA10*	A10	Marker798064 – Marker800511	12.71–13.68	214	2,476/62	963/71
*FTC05*	C05	Marker1282614 – Marker1284391	1.21–1.64	130	1,787/29	1,014/52

Chr., chromosome; NO., number.

a The gene number confirmed by the Darmor-bzh genome in the target intervals.

b Among “a” genes, the number of genes that possess SNPsb in the target intervals.

c Among “a” genes, the number of genes that possess InDelsc in the target intervals.

### Comparison of the Flowering-Time Loci Between the Present and Previous Confidence Intervals

**Figure 3:**
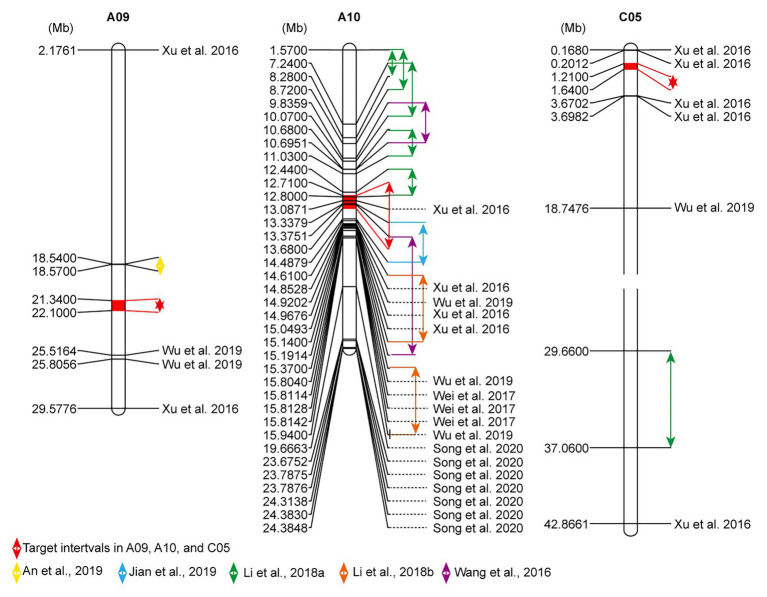
Comparison of flowering-time sites of A09, A10, and C05 chromosomes between current and previous studies. The physical locations related to flowering time were arrayed by MapChart 2.2 software. The highlighted fill segments with red double arrows expose the present target intervals. Other collected QTL intervals also are annotated by double arrows, and significantly linked single nucleotide polymorphisms (SNPs) with flowering time were noted with the black reference.

In order to validate the target intervals, the published loci associated with flowering time in *B. napus* were analyzed. In total, 344 Loci related to flowering time were extracted, which were distributed across almost all chromosomes of *B. napus*. A large number of the loci (254/344) located on A02, A03, A07, A10, C02, C06, and C09 (Supplementary Table S4). There were certain disparities in the loci of flowering time among different research backgrounds but within certain concordant intervals. The target interval *FTA10* overlapped with the confidence intervals detected in previous studies (Wang et al., 2016; Xu et al., 2016; Li et al., 2018a; Jian et al., 2019; Figure 3). *FTA09* and *FTC05* did not overlap with these loci. For *FTA09*, there was a distance of 2.77 and 3.42 Mb away from the loci reported by An et al. (2019) and Wu et al. (2019), respectively. For *FTC05*, which was 1.01–2.03 Mb away from the SNPs excavated by Xu et al. (2016; Figure 3). Therefore, it was speculated that these existed novel and reliable loci for flowering-time regulation in the two intervals.

### Candidate Genes for Flowering Time

According to the Darmor-*bzh* reference genome, a total of 448 genes occupied the three target regions were identified (Supplementary Table S5). To understand the genetic variations of these genes, the SNPs and InDels based on the whole-genome re-sequencing data of both parents (DZ and GW) were analyzed. In total, 2,759, 2,476, and 1,787 SNPs were detected in *FTA09*, *FTA10*, and *FTC05*, respectively. Correspondingly, 938, 963, and 1,014 InDels were also detected in these three intervals (Supplementary Table S6). In *FTA09*, 55 of 104 genes possessed SNPs or InDels. In *FTA10* and *FTC05*, 35.51% (76/214) and 42.31% (55/130) of genes contained SNPs or InDels, respectively (Table 4 and Supplementary Table S6). A total of 186 variant genes in these three target intervals were identified as the hypothesized candidate genes.

**Figure 4:**
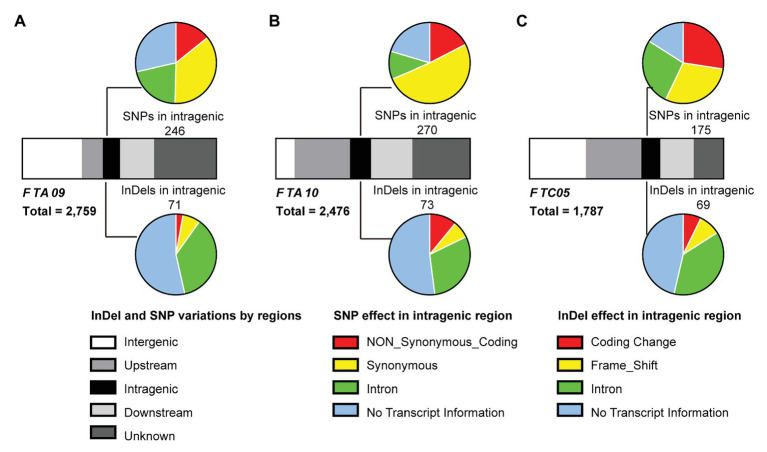
Analysis of SNPs and insertion-deletions (InDels) between both parents in the three target intervals, *FTA09* **(A)**, *FTA10* **(B)**, and *FTC05* **(C)**. Strip-shape charts show the distribution of SNPs and InDels in different genomic regions. The upstream and downstream represent the 5 kb within the region of transcription start and stop sites, respectively. Pie charts show the effects of SNP (upside) and InDel (underside) in the intragenic regions. And the corresponding quantity of SNP or InDel is labeled near the pie chart.

Among these variations, a substantial portion (61.81–76.90%) was located outside of the genes, including the scope within or beyond 5 kb upstream and downstream of the transcription start and stop sites. Only 8.57–9.97% variations were located in the intragenic region (Figure 4 and Supplementary Table S6). Non-synonymous variations with a percentage of 13.25–24.18% were found in the coding sequence among the intragenic region (Figure 4 and Supplementary Table S6). It was speculated that the key genes regulating flowering time existed in the target intervals from the mass of genetic variations between the parents.

**Figure 5:**
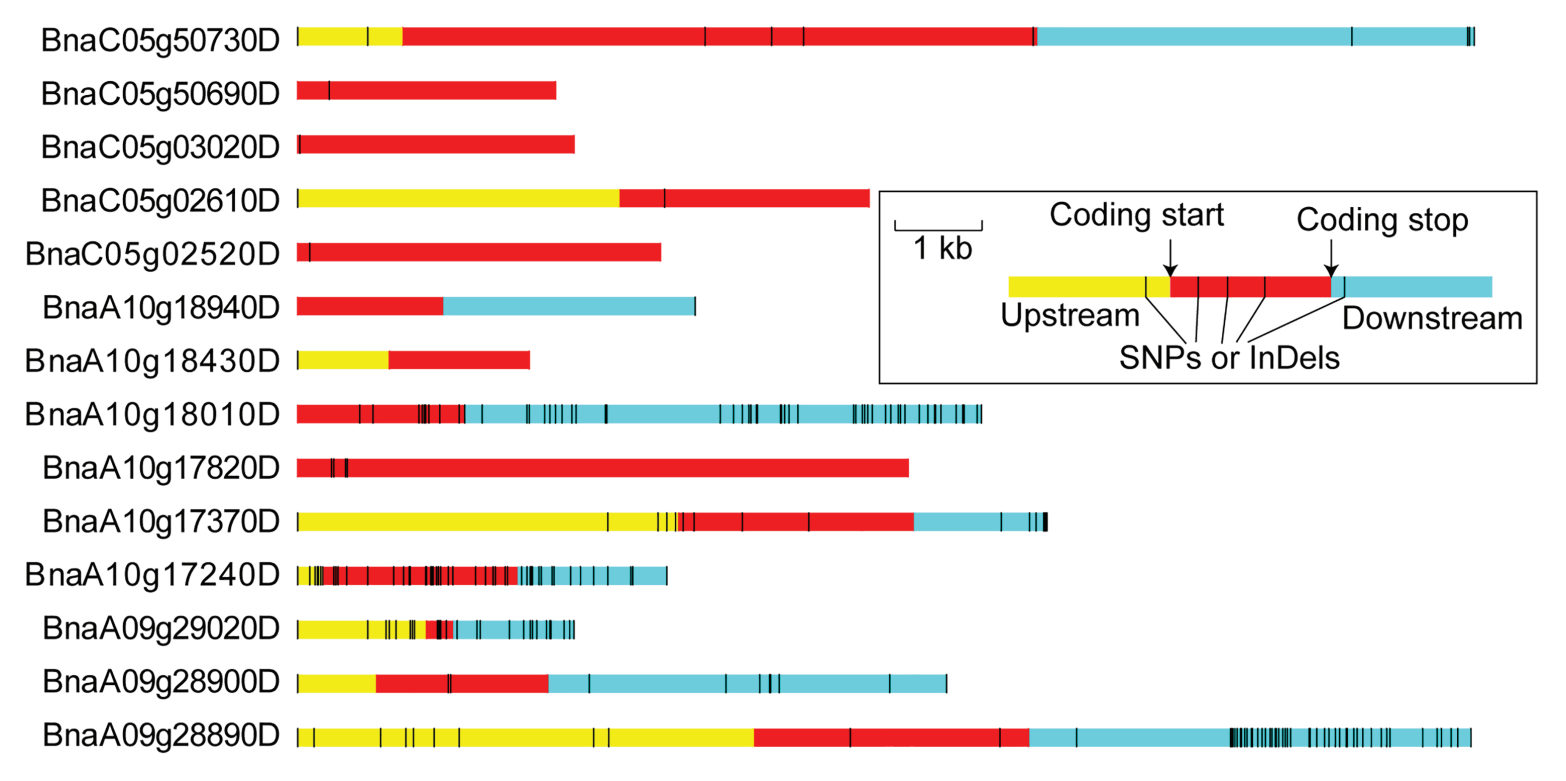
Variation distribution of the candidate genes. The upstream and downstream represent the 5 kb within the region of transcription start and stop sites, respectively. The regions are markers by different colors and the relative physical distance also are shown in the diagram.

To anchor the candidate genes responsible for the flowering time in *B. napus*, the functions of 186 variant genes were annotated basing on Pfam, InterPro, GO, and KEGG databases, among which, 90.32% were annotated (Supplementary Table S5). Fourteen genes were annotated with flowering-time regulation, including *RGA-LIKE PROTEIN 3* (*RGL3*) ortholog (*BnaA10g17240D*), *FRIGIDA LIKE 1* (*FRL1*) ortholog (*BnaA10g18010D*), *CONSTANS* (*CO*) ortholog (*BnaA10g18430D*) in *FTA10* and *CRYPTOCHROME 2* (*CRY2*) ortholog (*BnaC05g02520D*) in *FTC05*. Six of fourteen genes existed SNP or InDel variations in the upstream, intragenic region, and downstream between both parents; one and one gene had variations on the upstream and intragenic region, and intragenic region and downstream, respectively; and one, one, and four genes had SNP or InDel variations in the upstream, downstream, and coding region, severally (Figure 5). Among which, three genes (*BnaA09g29020D*, *BnaA10g17240D*, and *BnaA10g18010D*) had non-synonymous coding and seven genes existed variations within 2 kb region of transcription initiation (Supplementary Table S6).

### InDel Markers Linked to Flowering Time in the Target Intervals

**Figure 6:**
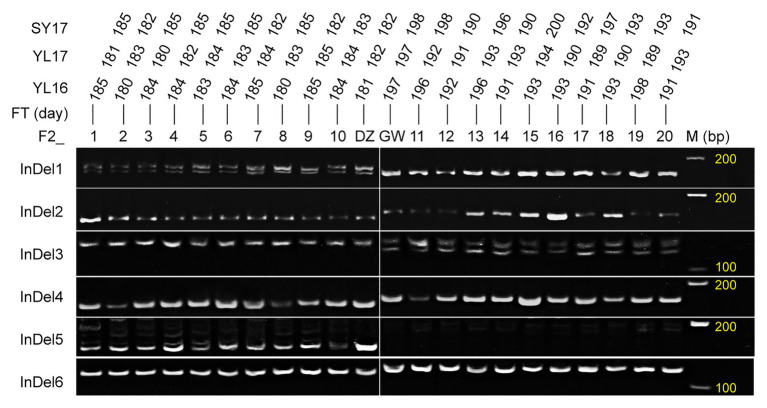
Validation of the developed InDel markers using the parents (female GW and male DZ) and extreme plants. F2_1–10 and 11–20 represent the early and late flowering plants that were screened out according to the flowering time of Yangling in 2016 (YL16), Yangling in 2017 (YL17), and Sanyuan in 2017 (SY17) among the F_2a_ population.

Six InDel markers were developed based on the re-sequencing data (Supplementary Table S3). The markers InDel1 and InDel2 were from the genes *BnaA09g28720D* and *BnaA09g28740D* in the *FTA09* region, respectively. InDel3 and InDel4 were derived from *BnaA10g17220D* and *BnaA10g18220D* on *FTA10*, respectively. InDel5 and InDel6 were from *BnaC05g02520D* and *BnaC05g02490D* on *FTC05*, respectively. All InDel markers showed polymorphism between the two parents and 20 extreme plants from F_2a_ (Figure 6). The results indicated the six InDel markers in the three target intervals were associated with the flowering time, and these candidate genes around the six InDel markers were reliable to some extent.

## Discussion

### Construction of the High-Density Genetic Map for QTL Mapping

The SSR is a class of traditional molecular markers for QTL mapping (Smith et al., 1997; McCouch et al., 2002). Despite it is easily used for gene mapping and tagging, it has limited potential in practical plant breeding, because it is difficult to discern precisely multiple alleles and integrate SSR data from different backgrounds. Moreover, the distribution of SSR in plant genomes is not enough for fine mapping (Piquemal et al., 2005; Xu and Crouch, 2008). In this study, only 107 of 1,030 pairs of SSR primers showed polymorphism among 170 F_2a_ population and both parents. A sparse linkage map with an average density of 8.5 cM was constructed using these SSR loci (Supplementary Table S1). Based on this map, the QTLs for FT with the range from 12.71 to 44.24 cM were identified preliminarily (Table 2 and Supplementary Figure S2). The wide range of QTLs makes it difficult to anchor the candidate genes associated with flowering-time regulation. Therefore, it is necessary to narrow the QTL interval, and one of the most effective methods is to increase the marker density of the QTL interval. The high-density genetic linkage map is a very useful tool to improve the accuracy of QTLs. In recent years, SNP as a class of ideal marker, has been extensively used as an effective tool to construct high-density maps basing on next-generation sequencing techniques (Liu et al., 2013; Zhou et al., 2018; Jian et al., 2019). SLAF-seq technology, a cost-effective platform, is recently developed for SNP exploitation, domestication analysis, high-density linkage map construction, genome-wide linkage analysis, and association analysis of agronomic traits (Sun et al., 2013; Zhou et al., 2017; Zheng et al., 2019; Ren et al., 2020). This technology was adopted in our study, through which, 3,356 high-quality SNPs were obtained, and a high-resolution genetic map was constructed (Supplementary Table S2 and Supplementary Figure S3). The average distance between consecutive markers was only 0.70 cM (Supplementary Table S2). Basing on the high-density map, 14 QTLs were detected, and all of the QTL intervals were less than 2 Mb (Figure 1 and Table 3).

### Identification and Evaluation of Target Intervals for Flowering-Time Loci

In order to narrow the interesting intervals related to flowering time, an IH-DGM was established by integrating the SSR loci linked to FT into the HRGM. On the IH-DGM, a co-localized interval *FTA10* on A10 was detected. *FTA10* was an overlapping region among three QTLs (*qFTSY17-7*, *qBUTYL18-A10*, and *qBOTYL18-A10*) that were detected in SY17 and YL18 (Figure 2). The target interval can be considered as one stable QTL, because the involved QTLs *qBUTYL18-A10*, and *qBOTYL18-A10* detected in YL18 had an adjacent distance (only 3.66 cM) in the HRGM (Figure 1 and Table 3), and *qFTSY17-7* detected in SY17 also overlapped with the two QTLs. Besides, *FTA10* overlapped with previous QTLs related to flowering time (Figure 3). However, the PV explained by previous QTLs were lower than 10% (Wang et al., 2016; Xu et al., 2016; Li et al., 2018a; Jian et al., 2019). In this study, the three QTLs involved in *FTA10* accounted for high PV (all over 10%, Tables 2 and 3). Generally, environmentally stable QTLs with high PV provide a higher probability to identify the major genes. These results led us to believe that there exist major genes responsible for flowering-time regulation in *FTA10*.

However, there were no co-localized intervals between SSR- and SLAF-based QTLs on the integrated A06 and C05. On A06, *qBOTYL18-A6* detected in YL18 was mapped to the region of 1.36–2.05 cM, and the QTL *qFTYL16-2* detected in YL16 was integrated into the region of 50.56–73.76 cM. Four QTLs detected in YL16 and YL18 based on the SSR and SLAF genetic map, were mapped onto different loci on the integrated C05, *qBUTYL18-C5*, and *qBOTYL18-C5* resided in the region between 15.66 and 20.39 cM, *qFTYL16-5* was mapped onto the region between 99.60 and 129.51 cM, and *qFTYL18-C5* was located in the regions of 165.32–172.55 cM (Figure 2). It was speculated that the results were due to the gap between SSR and SLAF markers. The subsequent development of more polymorphic markers is needed to confirm whether stable and reliable loci exist on the two chromosomes.

Beyond the environmentally QTL, in view of the significant correlation among FT, BUT, and BOT traits in YL18 (Supplementary Table S7), these overlapped QTLs among the three traits were also selected as the interesting intervals. Two fully overlapping intervals (*FTA09* and *FTC05*) between BUT and BOT were confirmed. The phenotypic variations of all four QTLs (*qBUTYL18-A9*, *qBOTYL18-A9*, *qBUTYL18-C5*, and *qBOTYL18-C5*) involved in *FTA09* and *FTC05* were over 10% (Tables 3 and 4). In *FTA09*, there were no overlapping loci with previous QTLs collected in this study (Figure 3). We speculate that there have novel loci associated with flowering time in the interval. On C05, we narrowed the regions identified by Xu et al. (2016) and anchored the interesting interval to a 430-kb region (Figure 3). The result may guide that we easily identify the flowering genes in C05.

### Candidate Genes Analysis for Flowering-Time Regulation

The QTLs for flowering time vary under different genetic backgrounds and conditions. At present, the loci related to flowering time were detected in almost all chromosomes in rapeseed (Wang et al., 2016; Xu et al., 2016; Wei et al., 2017; Li et al., 2018a,b; Shah et al., 2018; Shen et al., 2018; An et al., 2019; Jian et al., 2019; Raman et al., 2019; Wu et al., 2019; Song et al., 2020), which increased the difficulty of gene cloning and functional analysis for flowering-time traits in rapeseed. Most of the previous studies about the genetic mechanisms have been focused on a number of central flowering genes, such as *FLC* (Chen et al., 2018; Yin et al., 2020), *FT* (Wang et al., 2009; Guo et al., 2014), *CO* (Robert et al., 1998), and *FRI* (Wang et al., 2011). However, numerous flowering genes in rapeseed have not been excavated.

In this study, a novel region *FTA09*, two stable intervals *FTA10* and *FTC05* were identified (Table 4). Fourteen candidate genes with genetic variations between both parents were ascertained in the three target intervals according to functional annotations similar with flowering genes of *Arabidopsis* (Supplementary Table S5), including the homologs of *CO* (*BnaA10g18430D*), *FRL1* (*BnaA10g18010D*), and *RGL3* (*BnaA10g17240D*) in *FTA10*, and the ortholog of *CRY2* (*BnaC05g02520D*) gene in *FTC05*. Among these, *BnaA10g18010D* and *BnaA10g18430D* were also noted as key genes associated with flowering time in previous studies (Wang et al., 2016; Xu et al., 2016; Li et al., 2018a; Jian et al., 2019), but the functions need to be further studied. The other two homologous genes (*RGL3* and *CRY2*) have been reported to regulate flowering in *Arabidopsis* (Michaels et al., 2004; Shi et al., 2016; Liu et al., 2018). However, no associated functional study with flowering regulation has been reported in rapeseed. Six InDel markers linked to flowering time were developed around these candidate genes (Figure 6), indirectly demonstrating that the candidate genes and target intervals were trustworthy.

The genetic variations of flowering time genes are ubiquitous in different *B. napus* accessions. There were SNPs in the promoter regions of *FLOWERING LOCUS T* (*FT*) and *FLC* orthologs, corresponding to different rapeseed ecotype groups (Wu et al., 2019). Significant SNPs were found near *A. thaliana CO* orthologous gene *BnaC09g41990D* (Xu et al., 2016). These finds showed that genetic variations of flowering genes played important roles in adjusting flowering time. SNP and InDel variations were also found between the two parents in the genomic sequences of the 14 candidate genes, including the regions 5-kb downstream and upstream of the genes, and the coding regions (Figure 5 and Supplementary Table S6). It was also found that three genes (*BnaA09g29020D*, *BnaA10g17240D*, and *BnaA10g18010D*) occurred non-synonymous coding variations between the two parents, therefore, it was speculated that these genes maybe the key genes responsible for the flowering time. Cloning and functional analysis of these candidate genes should be conducted in the future, which will help to expound the regulatory mechanism of flowering time in *B. napus*.

## Conclusion

In conclusion, we identified three precise and reliable interested regions for flowering time in rapeseed by jointing analysis of the SSR and SLAF-seq across two F_2_ populations in four environments. Among of which, *FTA09* was considered as a novel interval. Fourteen candidate genes and six InDel markers linked to flowering time were identified in the three target intervals ulteriorly. These findings may serve as the foundation for genetic mechanism analysis of flowering time in *B. napus*.

## Resource Identification Initiative

SPSS (*SedDB*, RRID: SCR_002865), R package: lme4 (*SedDB*, RRID: SCR_015654), JoinMap (*SedDB*, RRID: SCR_009248), MapChart (*SedDB*, RRID: SCR_009273), GATK (*SedDB*, RRID: SCR_001876), Samtools (*SedDB*, RRID: SCR_002105), SMOOTH (*SedDB*, RRID: SCR_009398), Pfam (*SedDB*, RRID: SCR_004726), InterPro (*SedDB*, RRID: SCR_006695), GO (*SedDB*, RRID: SCR_002811), KEGG (*SedDB*, RRID: SCR_012773).

## Data Availability Statement

The original contributions presented in the study are publicly available. This data can be found at: NCBI, accession number – PRJNA675966.

## Author Contributions

AX and ZH conceived, designed, and supervised the research. YX performed the main data analysis, manuscript drafting, and revision. BZ, NM, and XiL finished the population construction, SSR-based mapping, and SLAF-seq. MQ, YZ, and KW conducted data collection and manuscript revision. NG, KZ, XnL, and MZ worked on the field material management and phenotypic survey. All authors contributed to the article and approved the submitted version.

### Conflict of Interest

The authors declare that they have no known competing financial interests or personal relationships that could have appeared to influence the work reported in this paper.

## Footnotes

^1^https://www.r-project.org/

^2^https://brcwebportal.cos.ncsu.edu/qtlcart/WQTLCart.htm

^3^http://www.genoscope.cns.fr/brassicanapus/

^4^http://pfam.xfam.org/

^5^http://www.ebi.ac.uk/InterPro/

^6^http://www.geneontology.org/

^7^http://www.genome.jp/kegg/
