# Supplementary material for: Quantitative Trait Locus Mapping and Identification of Candidate Genes Controlling Flowering Time in Brassica napus L
Source: Front Plant Sci. 2021 Feb 3;11:626205. doi: 10.3389/fpls.2020.626205 (PMC7886670; doi:10.3389/fpls.2020.626205)
Supplement: Supplementary file 1 [file Data_Sheet_1.zip › Supplementary Material.doc]

***Supplementary Material***

1 **Supplementary Tables**

**TABLE S1** Detailed information of SSR genetic map constructed in the F2a population.

**TABLE S2** Characteristics of the high-resolution genetic map.

**TABLE S3** Primer sequence of SSR and InDel markers linked to flowering time sites.

**TABLE S4** Collected loci associated with flowering time in B. napus from previous studies.

**TABLE S5** Information of genes within the three co-localized intervals.

**TABLE S6** SNPs and InDels in the three co-localized intervals.

**TABLE S7** Correlation coefficient among FT, BUT and BOT traits in YL.

2 **Supplementary Figures**

**FIGURE S1** Frequency distribution of flowering time associated traits in lines.

**FIGURE S2** The distribution of QTLs for flowering time in *B. napus* based on the SSR genetic map.

**FIGURE S3** Information of high-resolution genetic map.
