# Supplementary material for: Quantitative Trait Locus Mapping and Identification of Candidate Genes Controlling Flowering Time in Brassica napus L
Source: Front Plant Sci. 2021 Feb 3;11:626205. doi: 10.3389/fpls.2020.626205 (PMC7886670; doi:10.3389/fpls.2020.626205)
Supplement: Supplementary file 1 [file Data_Sheet_1.zip › Supplementary Figures.doc]

# Supplementary Figures

**Figure S1.** Frequency distribution of traits associated with flowering time in lines. Red, green, and blue colors show the flowering time (FT), budding time (BUT), and bolting time (BOT), respectively. YL16 and YL18 reprezb hsent F_2a_ and F_2b_ populations in Yangling in 2016 and 2018, YL17 and SY17 represent F_2:3_ families in Yangling and Sanyuan in 2017. Black and gray arrows represent DZ and GW respectively.

**Figure S2.** The distribution of initial QTLs for flowering time in *B. napus* based on the SSR genetic map. The number of linkage groups is shown at the top. The genetic distance (cM) and SSR markers are indicated to the left and right, respectively. The QTLs are labeled on the right of the linkage groups. The solid and diagonal blocks represent the QTLs detected from F_2a_ population and F_2:3_ families, respectively.

**Figure S3.** Information of high-resolution genetic map. **(A)** The high-resolution linkage map. The linkage group number is shown on the *x*-axis, and the *y*-axis shows the genetic distance (cM). The black bars in each linkage group represent the mapped SLAF-seq markers. **(B)** Collinearity analysis of the genetic map and genome. The abscissa is the genetic distance of each linkage group; the ordinate is the physical length of each linkage group, which scatters the form of markers in the genome and genetic map collinearity. Different colors represent different chromosomes or linkage groups.
